# Efficacy of capillary pattern type IIIA/IIIB by magnifying narrow band imaging for estimating depth of invasion of early colorectal neoplasms

**DOI:** 10.1186/1471-230X-10-33

**Published:** 2010-03-27

**Authors:** Hiroaki Ikematsu, Takahisa Matsuda, Fabian Emura, Yutaka Saito, Toshio Uraoka, Kuang-I Fu, Kazuhiro Kaneko, Atsushi Ochiai, Takahiro Fujimori, Yasushi Sano

**Affiliations:** 1 National Cancer Center East Hospital, Department of GI Oncology & Endoscopy, Chiba, Japan; 2 National Cancer Center Hospital, Endoscopy Division, Tokyo, Japan; 3 Advanced Digestive Endoscopy, Emura Center Latino America & Emura Foundation for the Promotion of Cancer Research. Universidad deLa Sabana, Medical School Bogotá, Colombia; 4 Okayama University Hospital, Department of Endoscopy, Okayama, Japan; 5 Juntendo University Nerima Hospital, Department of Gastroenterology, Tokyo, Japan; 6 National Cancer Center Research Institute East, Pathology Division, Chiba, Japan; 7 Dokkyo University School of Medicine, Department of Surgical and Molecular Pathology, Tochigi, Japan; 8 Sano Hospital, Gastrointestinal Center, Kobe, Japan

## Abstract

**Background:**

Capillary patterns (CP) observed by magnifying Narrow Band Imaging (NBI) are useful for differentiating non-adenomatous from adenomatous colorectal polyps. However, there are few studies concerning the effectiveness of magnifying NBI for determining the depth of invasion in early colorectal neoplasms. We aimed to determine whether CP type IIIA/IIIB identified by magnifying NBI is effective for estimating the depth of invasion in early colorectal neoplasms.

**Methods:**

A series of 127 consecutive patients with 130 colorectal lesions were evaluated from October 2005 to October 2007 at the National Cancer Center Hospital East, Chiba, Japan. Lesions were classified as CP type IIIA or type IIIB according to the NBI CP classification. Lesions were histopathologically evaluated. Inter and intraobserver variabilities were assessed by three colonoscopists experienced in NBI.

**Results:**

There were 15 adenomas, 66 intramucosal cancers (pM) and 49 submucosal cancers (pSM): 16 pSM superficial (pSM1) and 33 pSM deep cancers (pSM2-3). Among lesions diagnosed as CP IIIA 86 out of 91 (94.5%) were adenomas, pM-ca, or pSM1; among lesions diagnosed as CP IIIB 28 out of 39 (72%) were pSM2-3. Sensitivity, specificity and diagnostic accuracy of the CP type III for differentiating pM-ca or pSM1 (<1000 μm) from pSM2-3 (≥1000 μm) were 84.8%, 88.7 % and 87.7%, respectively. Interobserver variability: κ = 0.68, 0.67, 0.72. Intraobserver agreement: κ = 0.79, 0.76, 0.75

**Conclusion:**

Identification of CP type IIIA/IIIB by magnifying NBI is useful for estimating the depth of invasion of early colorectal neoplasms.

## Background

Following complete surgical resection it has been found that colorectal cancers confined to the intramucosal layer (pM) or invading less than 1000 μm into the submucosa (pSM1), with no lymphovascular invasion or signs of poor differentiated histology do not have lymph node (LN) metastasis. In contrast, lesions invading more than 1000 μm into the submucosa (pSM2-3) have a 6-12% LN metastatic rate [1-3]. Therefore, in vivo estimation of the depth of invasion in early colorectal lesions may be important for an adequate therapeutic strategy.

Several studies on the adenoma-carcinoma sequence have demonstrated a gradual increment in microvessel density and a reduction in the apoptosis process during the progression from low dysplasia to high dysplasia and cancer [4]. In addition it is well recognized that angiogenesis performs a critical role in the development of solid tumors [5,6] and that detailed characterization of lesions using advanced optical imaging techniques is possible. We therefore developed in the late nineties the NBI system as an in vivo approach for visualizing microvascular anatomy or microvessels morphologic changes in superficial neoplasia [7-9].

By using this narrow spectrum, contrast in the microvascular architecture on the surface of the lesions is markedly improved [10,11]. In accordance with our previous investigations, the microvascular architecture (capillary pattern: CP) was classified into three types (CP type I, II and III) [9,11,12]. Our observations demonstrated that the CP assessed by magnifying NBI is useful for differentiating small colorectal non-neoplastic from neoplastic polyps [13] and is highly accurate at distinguishing between low-grade dysplasia and high-grade dysplasia/invasive cancer, and thus can be used to predict the histopathology of colorectal neoplasia [14]. However, its usefulness in estimating the depth of invasion of early colorectal neoplasms (pM, pSM1 or pSM2-3) is still unclear. The aim of this study was to clarify the diagnostic accuracy of magnifying NBI for assessing the depth of invasion of T1 colorectal cancer.

## Methods

### Patients

A total of 127 consecutive patients with 130 lesions endoscopically diagnosed as NBI CP type IIIA/IIIB who underwent endoscopic or surgical resection at the National Cancer Center East Hospital (NCCEH) from October 2005 to October 2007 were analyzed. The protocol was approved by the medical ethics committee of our hospital, and written informed consents for diagnosis and treatment were obtained from all patients prior to the procedures. The study was performed in accordance with the ethical principles that have their origin in the Declaration of Helsinki. Cases judged as NBI CP III but with familial adenomatous polyposis (FAP), and inflammatory bowel disease (IBD) were excluded from the study. CP type III lesions with an obvious appearance of advanced cancer were also excluded.

### Colonoscopy procedure using the RGB sequential illumination based NBI system

Bowel preparation consisted of 2 to 3 L of polyethylene glycol solution in the morning before the procedure, as previously reported [15]. Hyoscine methobromide (10-20 mg IV) was administered if there were no contraindications, and light sedation with diazepam (3-5 mg IV) was used in selected subjects. All procedures were performed up to the cecum using high-definition colonoscopy (CF-H260AZI [with a magnifying power of 75 at maximum]; Olympus, Optical Co., Ltd., Tokyo, Japan) with NBI magnification. A videoendoscope system (EVIS LUCERA SPECTRUM; Olympus, Optical Co., Ltd., Tokyo, Japan) and a digital image filing system (nexus sif; Fujifilm, Tokyo, Japan) was used. In NBI mode using this system, the center wavelengths of the dedicated trichromatic optical filters are 540 and 415 nm, with bandwidths of 30 nm Optional enhancement setting was set at enhancement mode A5 and color mode 3. Lesions were classified macroscopically based on the Paris classification of superficial gastrointestinal lesions [2]. Next, lesions were observed in NBI and each CP were evaluated by magnifying NBI view in real time. For larger lesions, the highest quality NBI image from the macroscopically worst area (e.g. large nodule, depression and reddened area) was evaluated.

In lesions identified as CP type IIIA, snare polypectomy, endoscopic mucosal resection (EMR), or endoscopic submucosal dissection (ESD) were performed. In lesions identified as CP type IIIB, surgical or endoscopic resection was performed.

### Capillary pattern classification

**Figure 1:**
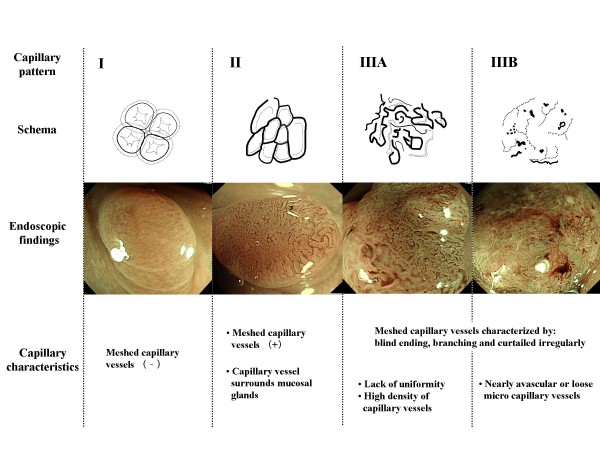
**Capillary pattern classification**.

Following conventional white light observation all cancer lesions were evaluated by magnifying NBI. Based on the surface characteristics of the meshed capillaries, CP type III were defined as demonstrating irregular and unarranged pattern in a mesh-like microvascular architecture and exhibiting at least one of the following: irregular size, complicated branching, disrupted irregular winding when compared to the regular small caliber capillaries observed in adenomatous polyps (CP type II) [Figure 1] [9,11,14]. Moreover, CP type III lesions were further classified into two groups: types IIIA or IIIB.

#### Capillary pattern type IIIA

**Figure 2:**
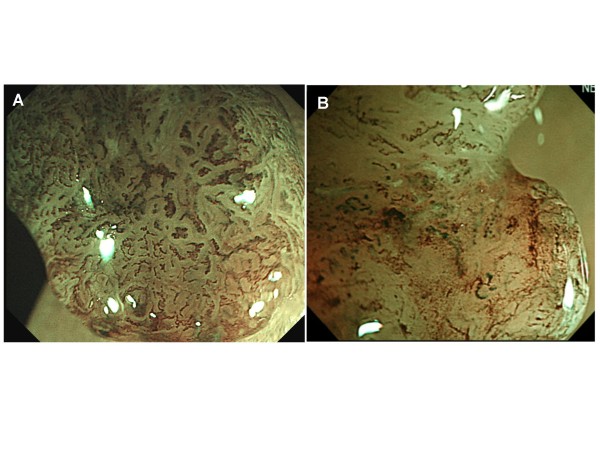
**Capillary pattern type IIIA, IIIB (magnifying NBI image at full max 75 times)**. A : Capillary pattern type IIIA. B : Capillary pattern type IIIB.

CP type III lesions clearly show visible microvascular architecture and high microvessel density with lack of uniformity, blind ending, branching and curtailed irregularly. [Figure 2A].

#### Capillary pattern type IIIB

CP type III lesions show a clear distinction between normal/cancerous mucosa on the surface (demarcated area) and the presence of a nearly avascular or loose microvascular area. [Figure 2B]

### Histological examination

All resected specimens were retrieved and immediately fixed in 10% buffered formalin solution and examined histologically using hematoxylin and eosin staining. Histopathological diagnosis was determined according to the Vienna classification [16]. Non-pedunculated lesions with a vertical invasion length of less than 1000 μm in the submucosal layer were classified as pSM1, and those with invasion of more than 1000 μm were classified as pSM2-3 [2]. Pedunculated lesions were categorized according to Haggitt's classification [17]. Pedunculated lesions with head invasion were classified as pSM1, and those with stalk invasion were classified as pSM2-3.

### Image evaluation

In an independent sub-study, inter- and intraobserver variabilities of the NBI CP type III for estimating the depth of early colorectal cancer were assessed by three colonoscopists experienced in NBI (YS, TM, HI). All 130 lesions were evaluated. The best magnifying NBI image of each lesion was selected. All selected images were arranged randomly for pattern assessment by the three readers who were blinded to the histological diagnosis of the lesions. All readers diagnosed the image of one pattern one day, and diagnosed another pattern one week later. The obtained data was not used for evaluating diagnostic accuracy of the lesions.

### Clinical data evaluation

The sensitivity, specificity, positive predictive value (PPV) and negative predictive value (NPV) of the CP type III for estimating the depth of invasion of early colorectal cancer was calculated according to the pathological report. Inter and intraobserver variabilities were calculated using kappa statistics.

## Results

### Clinicopathologic features of colorectal lesions

A total of 130 early colorectal lesions in 127 patients were analyzed. The clinicopathological data is shown in Table 1. According to the macroscopic types, there were 85 (65.4%) flat elevated and depressed lesions and 45 (34.6%) polypoid and protruded lesions. The mean lesion size was 17 mm (range 5-80 mm). There were 81 (62.3%) lesions located in the left colon and rectum and 49 (37.7%) lesions located in the right and transverse colon. Histologically, there were 15 adenomas, 66 pM, 49 submucosal cancers (pSM): 14 pSM1 and 33 pSM2-3. Among lesions diagnosed as CP IIIA 86 out of 91 (94.5%) were adenomas, pM, or pSM1; while among lesions diagnosed as CP IIIB 28 out of 39 (72%) were pSM2-3.

**Table 1:** Clinicopathological features of CP III lesions

No. of patients/lesions	127/130
Sex (Male/Female)	81/46
Mean age (y [range])	65.3 [41-86]
Macroscopic types	
Flat, depressed	85
Sessile, protruded	45
Mean size of lesions (mm [range])	17.0 [5-80]
Locations	
Right colon	49
Left colon, rectum	81
Histopathology	
Adenoma	15
pM*, pSM-superficial (pSM1)**	82
pSM-deep(pSM2-3)^#^	33

* intramucosal cancer, ** SM superficial invasion (<1000 μm), ^# ^SM deep invasion (≥1000 μm)

### Diagnostic accuracy, NPV and PPV of CP type IIIA and type IIIB

Sensitivity, specificity and diagnostic accuracy of the CP type IIIA/IIIB for differentiating pM or pSM1 (<1000 μm) from pSM2-3 (≥1000 μm) were 84.8%, 88.7% and 87.7%, respectively. The accuracy of CP type IIIA (NPV) was 94.5% (86/91), and that for lesions of CP type IIIB (PPV) was 71.8% (29/39) [Table 2].

**Table 2:** Sensitivity, specificity and diagnostic accuracy of the CP Type III

	Histological diagnosis	
	
	M*, SM-superficial (SM1)**	**SM-deep(SM2-3)**^**#**^
CP type IIIA	86	5
CP type IIIB	11	28

Sensitivity: 84.8%, Specificity: 88.7%, Accuracy: 87.7%,

NPV (negative predictive value): 94.5%, PPV (positive predictive value): 71.8%

* intramucosal cancer, ** SM superficial invasion (<1000 μm), ^# ^SM deep invasion (≥ 1000 μm)

### Image evaluation

The calculated interobserver variability of HI-YS, HI-TM, and YS-TM was κ = 0.68, 0.67, and 0.72, respectively. Intraobserver agreement of HI, YS, and TM was κ = 0.79, 0.76, 0.75, respectively (Table 3).

**Table 3:** Interobserver and intraobserver variabilities. (κ-value)

	HI-YS	HI-TM	YS-TM
Interobserver variabilities	0.68	0.67	0.72

	**HI**	**YS**	**TM**

Intraobserver variabilities	0.79	0.76	0.75

## Discussion

We previously demonstrated that NBI with magnification is a simple and reliable method to differentiate non-adenomatous from adenomatous colorectal polyps less than 10 mm (sensibility 96%, specificity 92, overall accuracy 95) [13] and, low grade adenomatous polyps from high grade adenomas or early colorectal neoplasms (Sensitivity 90%, specificity 97, overall accuracy 95) [14].

Based on the clinical observation and detailed characterization of lesions based on changes in the pattern and size of microvessels using magnifying NBI, we have described three different types of CP: CP type I (non-neoplastic lesion), CP type II (adenomatous lesion) and CP type III (cancerous lesion) [9]. The initial studies on CP type III lesions showed that within this group, there were lesions invading the intramucosal or the superficial submucosal layer, which require endoscopic treatment and lesions invading deeply into the submucosal layer, which require surgical treatment. These two subgroups could be differentiated from each based upon their respective CP patterns [17,18]. Concurrent to this study, we performed a pilot study using magnifying NBI to predict the depth of invasion of early colorectal lesions at the National Cancer Center Hospital, Tokyo. From the results of this investigation the following factors were found significantly more frequently in pSM2-3 lesions compared to pM-pSM1 lesions (P < 0.001): wide caliber, irregular caliber, tortuousity, irregularity, short length and non-dense arrangement. Multivariate analysis, however, revealed that irregularity and non-dense arrangement remained as independent factors [19]. These results supported the reliability of our classification. Consequently, we evaluated the efficacy of subdividing CP type III lesions into two groups (CP type IIIA/Type IIIB) and demonstrated that this may provide an effective in vivo method to predict the depth of invasion of colorectal neoplasms.

In this study, the overall diagnostic accuracy of the CP type IIIA classification to differentiate pM or pSM1 from pSM2-3 (87.7%) was quite similar to results obtained by magnifying chromocolonoscopy (87%) [20]. On the other hand, the sensitivity of using CP IIIA/IIIB to differentiate pM/pSM1 from pSM2-3 lesions (84%) was quite similar when compared to that obtained by the non-invasive/invasive pattern using MCC (85%) [21]. The specificities however, differed markedly (88% and 99%) in these two studies. Possible reasons for these differences are the inclusion of more than 3000 thousand adenomatous lesions in the study and due to the learning curve for estimating depth using NBI in early colorectal neoplasms.

When the NBI results were analyzed, it was found that 5 out of 91 (5.5%) lesions judged as CP type IIIA were ultimately classified as pSM2-3 in the pathological report. On the other hand, 11 out of 39 (28.2%) lesions diagnosed as CP IIIB were demonstrated to be pM or pSM1 according to the pathological report. Therefore the 71.8% positive predictive value (PPV) of CP was lower than the 86.5% PPV associated with using the pit pattern classification [21]. However diagnosis using pit pattern classification is time consuming due to the need to spray indigo carmine and crystal violet. An advantage of NBI is the ability to diagnose lesions without using any dye solution. Fundamentally, it is suggested that the lesion showing CP type IIIA is recommended for endoscopic treatment. In contrast, when a lesion is classified as CP type IIIB it is then necessary to perform Kudo's pit pattern observation using dye method or EUS assessment. Consequently, accurate pit pattern analysis and sufficient skills in magnifying colonoscopy are basic fundamentals required for accurate NBI diagnosis of depth of invasion in colorectal lesions [22].

In the sub-study, the rate of diagnostic agreement among the three observers was not excellent but good without variability (according to inter and intraobserver agreement rates). Some difficulties may relate to the study design in which the assessment was undertaken using only one image per lesion making the judgment difficult. Huang et al. reported a mean kappa value for inter and intraobserver agreement rate using pit pattern analysis of 0.716 and 0.810, respectively [23]. Considering that analysis of pit pattern has been performed for many years, the inter and intraobserver agreement rates associated with NBI reported in this study may indicate acceptable results. However, further multicenter research with endoscopists of different abilities and interobserver and intraobserver variability studies are necessary to validate these results.

The primary limitation of this study was that the NBI CP appearance was judged by a single endoscopist well experienced in magnifying NBI colonoscopy. Another point worth mentioning is that endoscopic judgment of the interobserver and intraobserver studies was carried out by experienced examiners. This means that the effectiveness of classifying CP by NBI deserves further validation studies including less experienced endoscopists.

## Conclusions

This study has demonstrated that the CP (Type IIIA/Type IIIB) evaluated by magnifying NBI may be an effective in vivo alternative method to predict the depth of invasion of colorectal neoplasms without the application of any dye solution. However, additional comparative research with MCC may be necessary to validate the results of this study.

## Competing interests

The authors declare that they have no competing interests.

## Authors' contributions

The study was planned by HI, TM, FE, YS, TU, K-IF, KK, YS participated in the design and coordination of the study. OA and TF analyzed a pathologic finding. HI collected the clinical data and wrote the manuscript. HI, TM and YS performed the statistical analyses. All authors have read and approved the final the manuscript.

## Pre-publication history

The pre-publication history for this paper can be accessed here:

http://www.biomedcentral.com/1471-230X/10/33/prepub
